# CD95 Is Part of a Let-7/p53/miR-34 Regulatory Network

**DOI:** 10.1371/journal.pone.0049636

**Published:** 2012-11-14

**Authors:** Annika Hau, Paolo Ceppi, Marcus E. Peter

**Affiliations:** Feinberg School of Medicine, Division Hematology/Oncology, Northwestern University, Chicago, Illinois, United States of America; University of Illinois at Chicago, United States of America

## Abstract

The death receptor CD95 (APO-1/Fas) mediates apoptosis induction upon ligation by its cognate ligand CD95L. Two types of CD95 signaling pathways have been identified, which are characterized by the absence (Type I) or presence (Type II) of mitochondrial involvement. Micro(mi)RNAs are small noncoding RNAs that negatively regulate gene expression. They are important regulators of differentiation processes and are found frequently deregulated in many human cancers. We recently showed that Type I cells express less of the differentiation marker miRNA let-7 and, hence, likely represent more advanced tumor cells than the let-7 high expressing Type II cells. We have now identified miR-34a as a selective marker for cells that are sensitive to CD95-mediated apoptosis. Both CD95 and miR-34a are p53 target genes, and consequently, both the sensitivity of cancer cells to CD95-mediated apoptosis and the ability to respond to p53 mediated DNA genotoxic stress are linked. Interestingly, while miR-34a was found to positively correlate with the ability of cells to respond to genotoxic stress, let-7 was negatively correlated. The expression level of CD95 inversely correlated with the expression of let-7 suggesting regulation of let-7 expression by CD95. To test a link between p53 and miR-34a, we altered the expression of CD95. This affected the ability of cells to activate p53 and to regulate miR-34a. Our data point to a novel regulatory network comprising p53, CD95, let-7, and miR-34a that affects cancer cell survival, differentiation, and sensitivity to apoptotic signals. The possible relevance of this regulatory network for cancer stem cells is discussed.

## Introduction

CD95 (Fas, APO-1, TNFRSF6) is a prototypical member of the TNF-receptor superfamily [1], [2]. CD95 belongs to the death receptors (DR), a group of cell surface receptors characterized by a conserved region in their cytoplasmic tail termed the Death Domain (DD). Like other DRs such as TNF-R1 and TRAIL receptors, CD95 is capable of mediating apoptosis induction in response to binding of its extracellular ligand, CD95L (CD178, FasL, TNFSF6) [3]. CD95L is expressed both as a membrane bound and a soluble form in various tissues with high expression in activated T lymphocytes and thymocytes [4], [5]. Most human cells are resistant to CD95-mediated apoptosis [3], but CD95 – CD95L signaling is important for elimination of virally infected and oncogene transformed cells, and it is pivotal in curbing autoimmune reactions [6]. The CD95 DD is able to interact and tether the adaptor molecule FADD which recruits caspase-8 leading to the formation of the death inducing signaling complex (DISC) and the activation of caspase-8 [7], [8]. In Type I cells such as T lymphocytes ample amounts of active caspase-8 are generated at the DISC for direct cleavage and subsequent activation of effector caspase-3. Coordinated release of mitochondrial proapoptotic contents may occur but is not necessary for completion of the apoptotic process. Importantly, expression of antiapoptotic Bcl-2 and Bcl-x_L_ cannot inhibit the ensuing death. However, in Type II cells such as hepatocytes and pancreatic island β-cells, a reduced amount of DISC is formed leading to weak activation of caspase-8. To induce apoptosis in these cells, mitochondrial amplification of the death signal is necessary. Release of mitochondrial proapoptotic factors such as Smac/Diablo and cytochrome c activates Apaf-1 generating enough active caspase-3 for apoptosis to proceed. In Type II cells, expression of Bcl-2 or Bcl-x_L_ inhibits the release of mitochondrial proapoptotic molecules and suppresses the apoptotic stimulus [7].

During the past decade the view that CD95 only signals death has been challenged by data showing that CD95 also activates proliferative and pro-survival pathways. When death is inhibited in Type II cells by Bcl-2 and Bcl-x_L_, the prosurvival factor NF-κB and the proproliferative ERK1/2, p38, AKT, and JNK pathways can be activated [3], [7]. In apoptosis resistant glioblastoma multiforme tumor cells, CD95 signaling activates the AKT/PI3K/GSKβ pathway by the Src-family protein Yes resulting in increased invasiveness, which is lost upon neutralization of CD95L [9]. In addition, we recently showed that CD95 signaling is critically required for cancer cell growth both in vitro and in vivo [10], thus suggesting a possible explanation as to why most tumor cells retain some CD95 expression despite the potential proapoptotic activity of CD95. In normal tissues, CD95 signaling has been shown to be required for regeneration and repair of the liver after partial hepatectomy, and this injury can protect hepatocytes from CD95 induced death [10], [11]. Finally, CD95 has been shown to possess pro-proliferative capabilities in neuronal stem cells [12]. A recent study assigned the pro-apoptotic signaling to the membrane-bound CD95L whereas the soluble ligand, sCD95L was devoid of apoptotic potential and was shown to promote development of autoimmune disorders and malignancy as evidenced by appearance of tumors in the liver [13]. In the context of cancer we previously proposed that Type II cells represent a more differentiated stage and Type I cells a less differentiated stage [14], [15]. Loss of differentiation and insensitivity to apoptosis are one of the hallmarks of cancer progression [16]. We postulated that when switching from Type II to Type I CD95 stimulation changes from being proapoptotic to mediating nonapoptotic signaling.

Micro(mi)RNAs are small, non-coding RNAs of 18–24 nucleotides capable of negatively regulating protein expression through either direct cleavage of their mRNA targets or repression of protein translation. miRNAs have emerged as master regulators of normal development, differentiation, and cell fate decisions, and are also shown to be involved in disease conditions such as cancer [17], [18]. This is not surprising as more than 800 miRNAs have been identified so far with estimates of them controlling more than 70% of the human genome [19]. We recently identified let-7 as a significant marker for Type II cells [15]. The let-7 family of miRNAs is a key regulator of embryogenesis and differentiation. Expression of let-7 is suppressed during embryogenesis and in ES cells but upregulated before birth and maintained at high levels during adulthood in most tissues [20]. We now demonstrate that expression of the p53-regulated miRNA miR-34a correlates with sensitivity of cells to CD95-mediated apoptosis. We have discovered a p53 regulated network that involves CD95, miR-34a, and let-7. Every component of this novel network has crucial functions in the generation or maintenance of cancer stem cells (CSCs). We suggest that CD95 suppresses the stemness-inhibitory let-7 family, thereby predisposing cells to possible adverse outcomes arising from the loss of this crucial maintainer of cellular differentiation. Let-7 targets and decreases CD95 expression through a negative feedback loop [21] that might subject cells to a loss of differentiation common during early steps of tumorigenesis [14], [20]. We also postulate a counteracting pathway in which CD95 maintains p53 expression and, indirectly, the expression of miR-34a, providing a substantial protective axis against the loss of let-7.

## Results

### miR-34a is a marker for cells sensitive to CD95-mediated apoptosis

**Figure 1:**
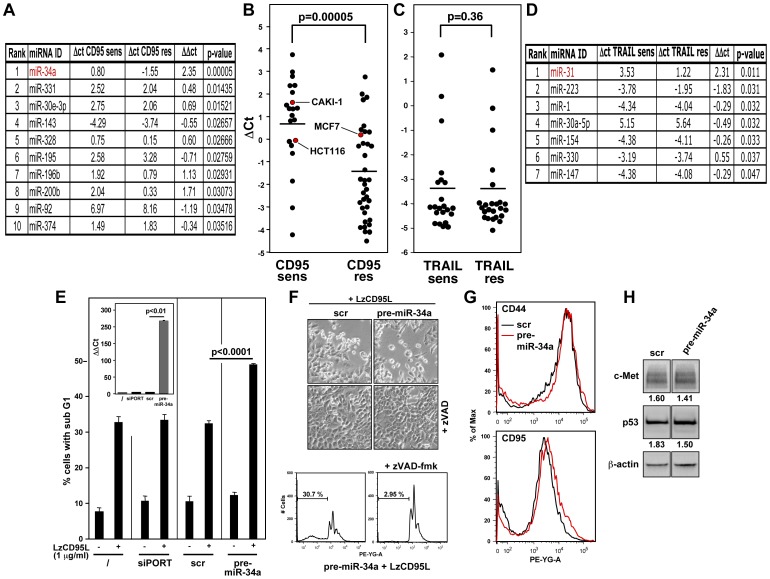
miR-34a is a marker for cells sensitive to CD95-mediated apoptosis. (*A*) Top 10 miRNAs differentially expressed between NCI60 cells sensitive and resistant to CD95-mediated apoptosis. (*B*) Dot plot showing miR-34a expression in 21 NCI60 cell lines sensitive and 36 cell lines resistant to CD95-mediated apoptosis. (*C*) Dot plot showing miR-34a expression in 33 NCI60 cell lines sensitive and 24 cell lines resistant to CD95-mediated apoptosis. (*D*) The miRNAs differentially expressed (p<0.05) between NCI60 cells sensitive and resistant to TRAIL-induced apoptosis. (*E*) HCT116 cells were either left untreated (/), transfection reagent treated (siPORT), or transfected with either scrambled pre-miR (scr) or pre-miR-34a and incubated with the indicated amounts of leucine-zipper tagged CD95 ligand (LzCD95L). Apoptotic cells were quantified 18 hrs after LzCD95L treatment. Inset shows real-time PCR analysis of miR-34a expression in the transfected cells. (*F*) Top: phase contrast image of HCT116 cells treated with scr, or pre-miR-34a and LzCD95L as described in C with and with out pretreatment with zVAD-fmk. Bottom: Sub-G1 peak analysis using PI staining and flow cytometry of HCT116 treated with pre-miR-34a and LzCD95L minus plus zVAD-fmk. (*G*) Surface staining of CD44 or CD95 in HCT116 cells 18 hours after transfection with either scr or pre-miR-34a. (*H*) Western blot analysis of c-Met and p53 in HCT116 cells three days after transfection with either scr or pre-miR-34a. Band intensities were quantified relative to actin for each lane.

In studying CD95-mediated apoptosis, our laboratory has made extensive use of a collection of 60 human cancer cell lines maintained by the National Cancer Institute's Developmental Therapeutics Program (NCI60). These cell lines represent nine different tissue origins. We have previously characterized 59 of the 60 cell lines for their sensitivity to CD95-mediated apoptosis [22]. Our analysis revealed that 22 of the 59 tested cell lines were sensitive to apoptosis upon stimulation of CD95, and that these could be separated into 11 Type I and 11 Type II cell lines. Interestingly, Type II cells were more sensitive to apoptosis induction when treated with soluble CD95L, and Type I cells were more sensitive when treated with the agonistic anti-CD95 antibody anti-APO-1. While let-7 was found to be a marker of differentiation in the NCI60 cells it did not correlate with the sensitivity of cells to CD95-mediated apoptosis even though signaling through CD95 was different between the two differentiation stages [15], [22]. To determine whether there are miRNAs that correlate with the sensitivity of cancer cells to CD95-mediated apoptosis, we interrogated an expression data set of 208 miRNAs determined by real-time PCR from the NCI60 cells used in our previous studies [15], [23]. Of 136 miRNAs that were detected in at least 30 of the 59 NCI60 cells, miR-34a most significantly correlated with CD95 apoptosis sensitive cells (**Figure 1A and B**). Interestingly, mir-34a expression did not significantly correlate with the sensitivity of cancer cells to apoptosis induced by LzTRAIL (**Figure 1C**) and the miRNA that most significantly correlated with TRAIL apoptosis sensitivity was miR-31 (**Figure 1D**). These data suggested that miR-34a is a selective marker for CD95 apoptosis sensitivity. In order to test whether an increase in miR-34a expression would cause an increase in the sensitivity of cells to CD95-mediated apoptosis, HCT116, which are moderately sensitive to CD95L induced apoptosis, were transfected with either a scrambled oligonucleotide or pre-miR-34a. Addition of leucine-zipper tagged CD95 ligand (LzCD95L) induced cell death in control transfected cells that was significantly enhanced in cells which had received pre-miR-34a (**Figure 1E**). This form of cell death was canonical caspase mediated apoptosis because it could be completely inhibited by pretreating cells with the pan caspase inhibitor zVAD-fmk (**Figure 1F**). These data suggested that miR-34a is not only a marker for cells sensitive to CD95-mediated apoptosis but that it can sensitize cells to this form of apoptosis. Sensitization was not due to an altered surface expression of CD95 or reduced expression of the miR-34a targets c-Met or CD44 or an increase in p53 expression (**Figure 1G and H**).

### CD95, p53, and miR-34a are functionally linked

**Figure 2:**
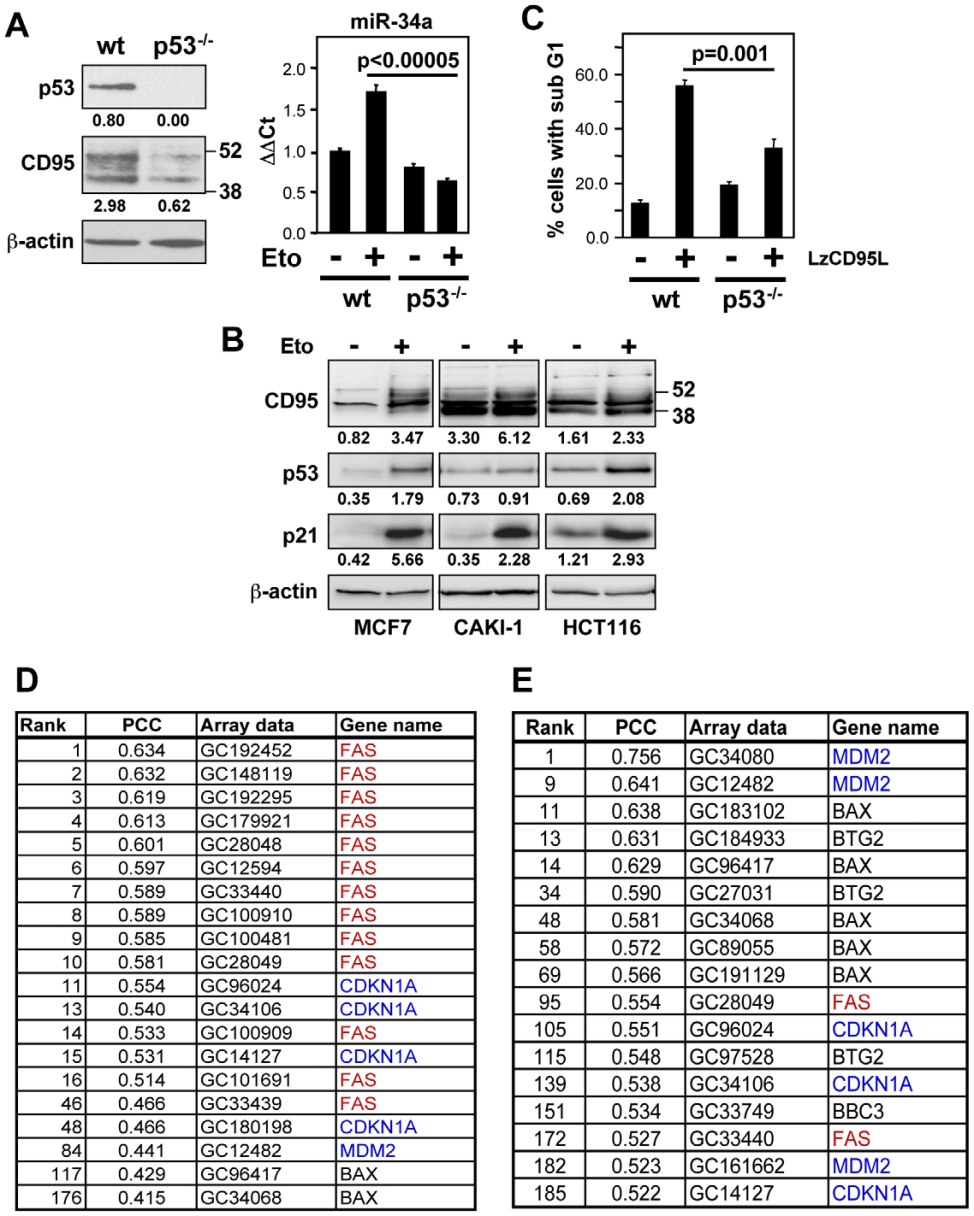
miR-34a and CD95 are p53 transcriptional targets that are functionally connected. (*A*) Western blot analysis of p53 and CD95 in HCT116 parental wild type (wt) and p53^−/−^ cells. For quantitative real-time PCR analysis of miR-34a, the same cells were treated with either control medium (−) or 10 µM etoposide (+) for 12 hrs. (*B*) MCF7, CAKI-1, and HCT116 cells were treated with control medium (−) or 10 µM etoposide (+) for 12 hrs and subjected to Western blot analysis. Band intensities were quantified relative to actin for each lane. (*C*) Apoptosis of HCT116 wt and p53^−/−^ cells upon LzCD95L treatment (1 µg/ml) for 18 hrs. (*D*) Key p53 targets positively correlating with the sensitivity of NCI60 cells to CD95-mediated apoptosis. (*E*) Genes whose expression positively correlates with p53 responsiveness in NCI60 cells.

Our finding that the expression of miR-34a positively correlates with the sensitivity of cells to undergo CD95-mediated apoptosis is in line with the proapoptotic function of both miR-34a and p53. In fact, p53 and CD95 are also functionally linked. CD95 contains an intra-intronic p53 enhancer, and is a bona fide p53 target gene [24] similar to miR-34a. HCT116 cells in which both p53 alleles have been knocked out (HCT116 p53^−/−^) express very little CD95 (**Figure 2A**) confirming the p53-dependency of CD95 expression. Consistent with this established connection between p53 activity and CD95 expression, treating cells with the DNA damaging reagent etoposide caused increased expression of p53, CD95, and the p53 target p21 in MCF7, CAKI-1, and HCT116 cells (**Figure 2B**). When HCT116 p53^−/−^ cells were treated with etoposide they were unable to upregulate miR-34a (**Figure 2A**), indicating that both miR-34a and CD95 upregulation are dependent on p53 expression. Additionally, when HCT116 p53^−/−^ cells were stimulated with LzCD95L they exhibited a decreased sensitivity to CD95-mediated apoptosis (**Figure 2C**).

miR-34a has been identified as a downstream effector of p53 mediating a number of its activities including apoptosis induction [25]–[29]. Together with the recognition that CD95 apoptosis sensitivity is in part regulated by the p53 status [30], the identification of miR-34a as a marker for CD95 apoptosis sensitive cells pointed at a connection between CD95 signaling and the p53 network. To determine whether sensitivity to CD95-mediated apoptosis and miR-34a expression in the NCI60 cells correlated with the expression of other known p53 targets we performed a COMPARE analysis [31] using the gene array data available on the NCI60 cells. Validating the analysis we found a strong correlation between the sensitivity to CD95-mediated apoptosis and the expression of CD95 (**Figure 2D**). In addition, we found that among the most highly correlating genes (of >18,000 genes analyzed) were a number of canonical p53 target genes including p21, MDM2, and BAX that were positively correlated with CD95 apoptosis sensitivity. A similar set of genes was found to positively correlate with the expression of miR-34a (data not shown). Because CD95 is, itself, a known p53 regulated gene, this points at a connection between CD95 apoptosis sensitivity, the expression of miR-34a, and a functional p53 pathway resulting in upregulation of p53 target genes; this is also consistent with the fact that p53 activation increases the surface expression of CD95 [32].

Only cancer cell lines expressing wild-type p53 have the potential to upregulate p53 regulated genes in response to genotoxic stress. However, even cells with wild-type p53 sometimes often acquire other mutations that result in a blunted p53 response. The functionality of the p53 pathway among the NCI60 cell lines, including both the p53 status as well as the response of all NCI60 cells to γ-irradiation, has been determined previously [33]. Among the NCI60 cell lines, 13 were found to express wild-type p53 and to respond to γ-irradiation with G1 arrest. To test the putative connection between p53 and miR-34a, we performed another COMPARE analysis comparing these 13 NCI60 lines with cell lines that did not respond to γ-irradiation with cell cycle arrest (**Figure 2E**). Again, expression of the same four p53 response genes correlated with the ability of p53 wild-type cells to respond with cell cycle arrest. Some receptors and signaling molecules are active in a seemingly constitutive fashion. Their “tonic” activity only becomes apparent when they are knocked down or knocked out [34]. Our data not only confirm the connection between p53 and CD95 but they also suggest that even without irradiation cells with an “active” p53 pathway are predisposed to respond with activation of p53 presumably due to tonic signaling through p53.

### CD95 expression affects the ability of cells to upregulate miR-34 in response to genotoxic stress

**Figure 3:**
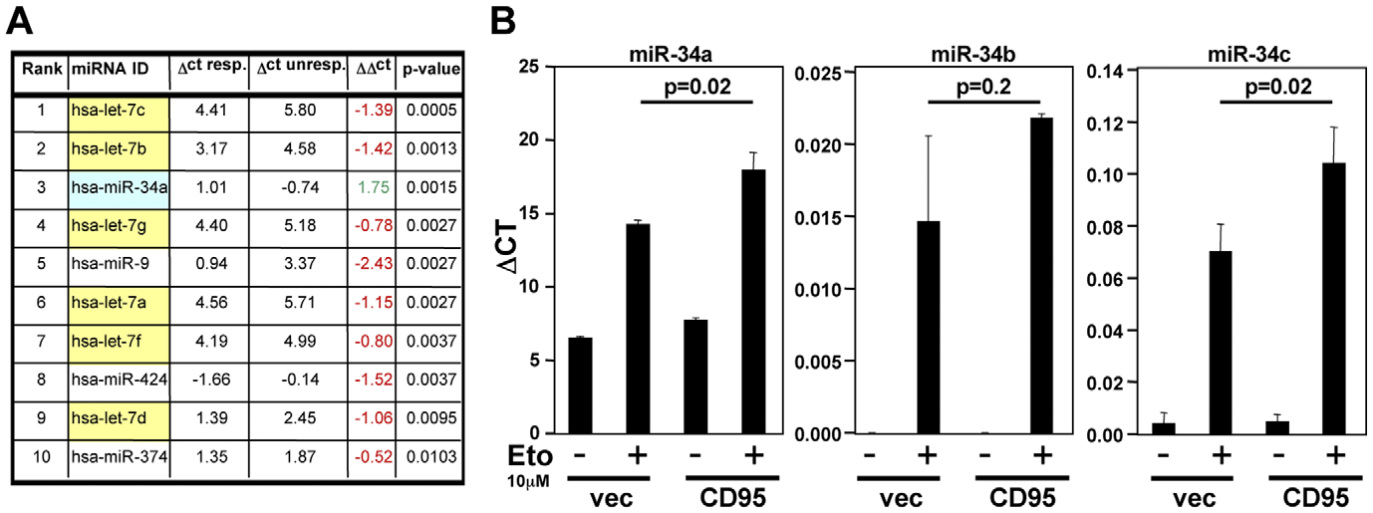
miR-34a and let-7 are functional opposites of p53-responsiveness. (*A*) Identification of the top 10 miRNAs differentially expressed between p53 responsive and unresponsive cells. All let-7 family members are highlighted in yellow and miR-34a in light blue. (*B*) Quantitative real-time PCR analysis of miR-34a, miR-34b, and miR-34c in MCF7 cells expressing either empty vector (vec) or CD95 (CD95) after 12 hr treatment with 10 µM etoposide.

**Figure 4:**
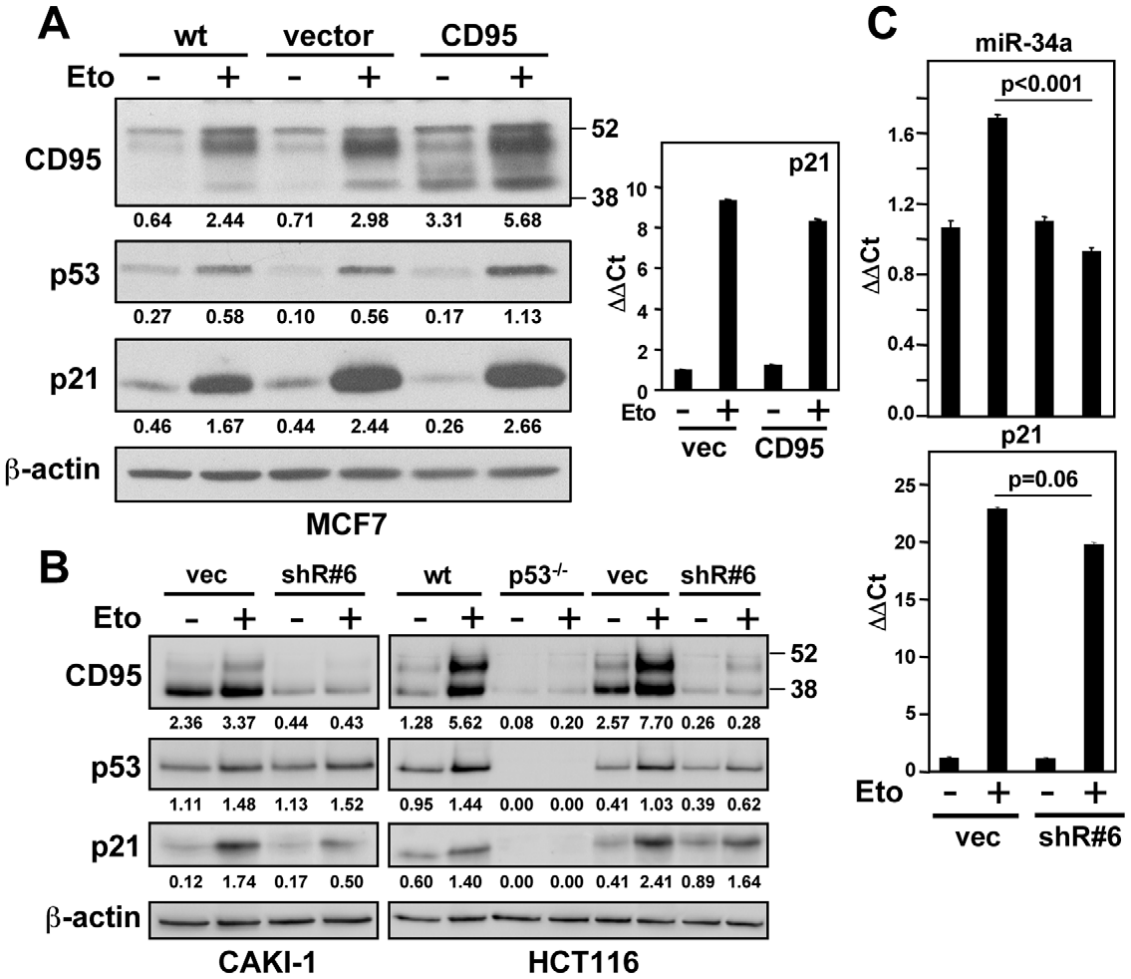
CD95 positively affects p53 expression. (*A*) Left panel: MCF7 parental, vector or CD95 expressing cells treated with 10 µM etoposide for 12 hrs analyzed by western blotting for CD95, p53, and p21 expression. Right panel: p21 mRNA upregulation after 12 hrs of 20 µM etoposide treatment in vector or CD95 transfected cells. (*B*) Left panel: Effect of CD95 knockdown with a lentiviral shRNA (shR#6) on etoposide induction in CAKI-1 cells. Right panel: Effect of CD95 knockdown on etoposide induction in HCT116 cells. In both panels cells treated with 10 µM etoposide for 12 hrs. Band intensities were quantified relative to actin for each lane. (*C*) miR-34a (upper panel) and p21 (lower panel) expression analysis by quantitative real-time PCR in CAKI-1 vector or CD95 knockdown cells using shR#6 after a 12 hrs treatment with of etoposide (10 µM).

Tonic signaling in cells with an intact p53 pathway was sufficient to allow detection of a correlation in expression of p53 with some of its target genes including CD95. In order to determine whether this also applied to miR-34a, we compared the expression of miRNAs significantly expressed in the NCI60 cells between p53 responder and nonresponder cells. miR-34a was the only miRNA that was significantly expressed more highly in the p53 responder cells (**Figure 3A** and data not shown). Because miR-34a was a positive regulator of the sensitivity to CD95-mediated apoptosis, we tested whether altering CD95 expression levels also affected the response of cells to stress induced by DNA damage (**Figure 3B**). Overexpression of CD95 in CD95 low expressing MCF7 cells caused a pronounced upregulation of miR-34a upon treatment with etoposide. Although miR-34b and miR-34c are minor species in these cells, these miRNAs also displayed an enhanced response to etoposide treatment in CD95 overexpressing cells (**Figure 3B**). Our data suggest that CD95 is not only a p53 target but it also contributes to the activity of p53 by either affecting p53 expression or stability, or by regulating p53 target genes independent of the status of p53. To test this we treated parental, vector expressing, and CD95 overexpressing MCF7 cells with etoposide to activate p53. A modest but reproducible increase in p53 expression was detected in the CD95 overexpressing cells (**Figure 4A**). In contrast, knock down of CD95 in either CD95 high expressing CAKI-1 or HCT116 cells, which resulted in reduced expression of CD95, attenuated the ability of the cells to respond to genotoxic stress by inducing p53, p21 or miR-34a (**Figure 4B and C**). These data suggest that CD95 directly regulates the expression of p53 and its ability to induce miR-34a expression.

### Let-7 expression inversely correlates with p53 signaling and expression of CD95

While miR-34a was the miRNA that best correlated with the ability of cells to respond to activation of p53, the most significant correlation between p53 responsiveness and the expression of miRNAs was a negative correlation with the let-7 family of miRNAs. Among the top ten miRNAs to most negatively correlate with p53 response status were 6 of the 9 distinguishable let-7 activities. This analysis suggested that miR-34a and let-7 inversely correlate with a p53 response which may directly affect the expression of CD95. Alternatively, the data do not exclude the possibility that CD95 regulates the expression of let-7.

**Figure 5:**
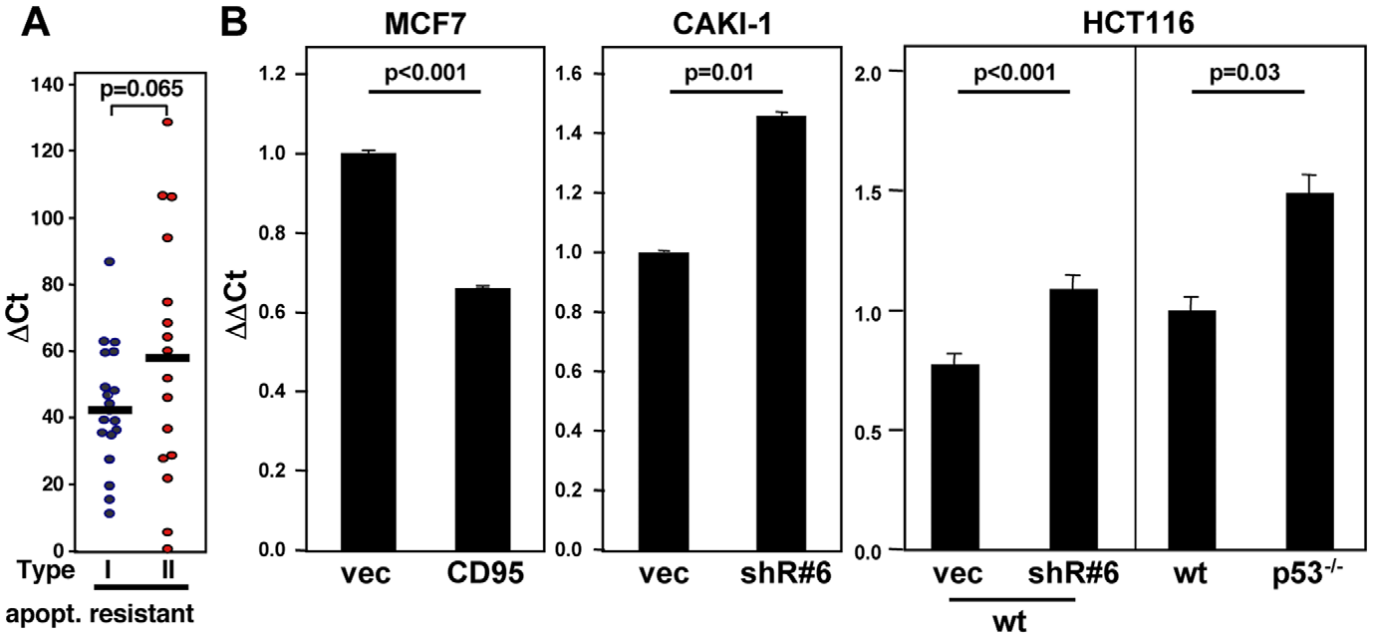
CD95 is a negative regulator of let-7. (*A*) Let-7d expression in apoptosis resistant Type I and Type II cells among the NCI60 cell lines. (*B*) Let-7c expression determined by quantitative real-time PCR in cells with modulated CD95 expression; left panel: MCF7 cells overexpressing CD95; center panel: CAKI-1 cells with CD95 knockdown with shR#6; right panel (left): HCT116 wt cells expressing either vector or shR#6, and (right) wt and p53^−/−^ cells.

It has been noted that expression of CD95 is higher in let-7 low Type I cells, and is lower in Type II cells with higher let-7 expression [35]. Consistent with these changes in cellular differentiation, we noticed that CD95 transfected Type II MCF7 cells exhibit a more mesenchymal morphology when overexpressing CD95 (unpublished observation). This hinted at variable CD95 signaling among cells of different differentiation stages, and also that CD95 signaling could regulate differentiation. While the lower expression of let-7 in the less differentiated cells was consistent with its function as a regulator of cellular differentiation, it was surprising that the difference in let-7 expression between the Type I and Type II cells that were sensitive to CD95-mediated apoptosis was more significant (p<0.0005, see our previous report [15]) than the difference in let-7 expression between the Type I and Type II cells that were completely resistant to CD95-mediated apoptosis (p>0.05, **Figure 5A**). This pointed at a situation similar to the link between p53 regulated genes and the ability of cells to respond to DNA stress as described above. Thus, the mere presence of CD95 may affect let-7 expression.

To test whether CD95 was a negative regulator of let-7 expression, we altered the expression of CD95 in different cell lines and monitored the expression of let-7. In MCF7 cells which express very low endogenous levels of CD95, ectopic expression of CD95 decreased the expression of mature let-7c (**Figure 5B**). A similar negative connection between CD95 and let-7 was also observed after knockdown of CD95 (using an shRNA targeting endogenous CD95 as previously described [10]) in CAKI-1 or HCT116 cells (**Figure 5B**). Interestingly, p53 deficient HCT116 cells expressing reduced CD95 (see **Figure 2A**) also showed increased let-7c expression (**Figure 5C**). Similar data were obtained for other let-7 family members including let-7g and d (data not shown).

## Discussion

### miR-34 is a selective marker for cancer cells that are sensitive to CD95-mediated apoptosis

In 2007, several groups identified the miR-34 family of miRNAs (miR-34a, b, and c) as a direct transcriptional target of the key tumor suppressor p53 [26]–[29], [36], [37]. miR-34a is ubiquitously expressed, whereas in most tissues miR-34b and miR-34c are minor species [37]. The importance of miR-34a and miR-34b is highlighted by their loss of expression in more than a dozen different cancers [37], [38] suggesting a crucial role for miR-34 in suppressing tumorigenesis. Not surprisingly, direct targets of miR-34a/b/c are enriched in genes controlling key cellular processes such as cell cycle control (cyclins D1, E2, and CDK4/6), cell proliferation (c-Myc, N-myc, E2F3, c-Met, Notch, Ezh2, HMGA2), and apoptosis (Bcl-2, SIRT1) [37], [39]. p53-independent expression of miR-34a has also been reported through activation of the Erk/Elk1 pathway [40].

We have now identified miR-34a as a marker of cancer cells that are more sensitive to CD95-mediated apoptosis, and as a sensitizer to apoptosis mediated by CD95. No correlation between expression of miR-34a was found with the sensitivity of cells to TRAIL-induced apoptosis. In contrast the miRNA that best correlated with sensitivity of cells to TRAIL induced apoptosis was miR-31 suggesting that the two death receptor families are connected differently to the network of miRNAs and their targets. This could be highly significant because conventional signaling components of death receptors so far have not allowed to explain how cancer cells can have highly different selective sensitivity to apoptosis induced by either CD95L or TRAIL [41]. One possible mechanism for the sensitization of cells to CD95-mediated apoptosis is suggested by data showing that miR-34 targets CD44, which has been shown to inhibit CD95-mediated apoptosis by directly binding the region required for CD95L engagement. Moreover a CD44 variant (CD44v6) binds the miR-34 target, c-Met, and this interaction is required for c-Met signaling [42]. However, we did not find altered expression of CD44 in miR-34a overexpressing cells. c-Met itself can sequester and inhibit CD95 activity through a similar mechanism, i.e., binding to the extracellular region required for ligand association [43]. However, we did not find evidence that c-Met is targeted in HCT116 cells. To further explore this aspect, we have compared the expression of c-Met with that of miR-34a in the panel of the NCI60 cells and a positive correlation, rather than a negative one, was found (data not shown). In addition, using an algorithm we recently developed to correlate gene with miRNA expression (summed (s)PCC) [44]) and using a web site we developed (miRConnect.org) we determined that c-Met mRNA is also slightly positively correlated with miR-34a using a data set based on real time PCR data of the miRNAs (miRConnect-Q). We recently extended the site to also include data sets from three primary cancers available at The Cancer Genome Atlas (TCGA) [45]. Again, we found strong positive correlation between c-Met expression and the expression of miR-34a in renal cancer and glioblastoma (sPCCs were 6.45 and 13.74, respectively). These analyses are not consistent with c-Met being a strong target for miR-34a in the context of cancer. The mechanism by which miR-34a sensitizes cells to CD95 mediated apoptposis is therefore currently unknown. mir-34a might regulate p53 expression by targeting SIRT1 which increases p53's acetylation, as recently shown [46].

Taken together, our data are consistent with a miR-34a-mediated increase in CD95 apoptotic signaling. We recently found that tumor cells expressing high levels of miR-200, which blocks epithelial-to-mesenchymal transition (EMT), are rendered more sensitive to CD95-mediated apoptosis [47] suggesting that during EMT tumor cells lose apoptosis sensitivity. Consistent with this finding, our analysis revealed that in tumor cells sensitive to CD95-mediated apoptosis the second most highly upregulated miRNA was miR-200b (miR-34a = 2.35 fold higher and miR-200b = 1.71 fold higher) (**Figure 1A**). These data suggest that the two miRNAs that regulate sensitivity to CD95-mediated apoptosis could be connected through their regulation of EMT. Consistent with this assumption are reports that describe both p53 and miR-34a as inhibitors of EMT [48], [49]. In fact, similar to miR-200 [23] overexpression of miR-34a alone can induce mesenchymal to epithelial transition (MET) in certain tumor cells [50].

### Connections between p53 and CD95

Multiple connections between CD95 and p53 have been described. Activated wt p53 induces both expression of CD95 mRNA and plasma membrane-associated CD95 [51], and this upregulation does not take place when p53 is mutated. Transcriptional induction of CD95 mRNA by p53 does not require de novo protein synthesis [52], and a p53-responsive element was identified in the first intron and three elements were identified in the CD95 promoter [24]. CD95 mRNA can also be induced by two p53 apoptosis deficient mutants [53], and the p53 homologues p63 and p73 through the intronic p53 response element [54]. This could possibly provide cells a fail-safe mechanism for the induction of apoptosis even in the absence of functional p53. An additional level of p53 regulation of CD95 has been shown to exist, which is independent of transcription, as cytoplasmic CD95 is trafficked from the Golgi to the cell surface upon DNA damage [32]. This relocation of CD95 results in a short term increase in CD95 apoptosis sensitivity, and does not occur in cells expressing a dominant negative p53. Our data now suggest that not only is CD95 a target of p53, but it also regulates the activity of p53. As shown in **Figure 4**, expression of CD95 negatively correlated with the ability of cells to mount a genotoxic stress response. Given our finding that CD95 expression inversely correlates with the expression of let-7 the possibility arose that changes in let-7 expression could regulate the amount of p53. We noticed that the p53 3′UTR contains a highly conserved seed match for let-7 (TargetScan 6.1) making this scenario a possibility. However, neither the overexpression nor the inhibition of let-7 had any effect on the expression of p53 in HCT116 cells, nor did fusion of the p53 3′UTR to a luciferase gene show any suppression when let-7 was coexpressed in 293T cells (data not shown). We therefore conclude that p53, at least in the tested cells, is not a target of let-7 suggesting that the effect of altered CD95 expression on p53 is independent of let-7.

A confounding problem complicating our studies is the fact that many of the connections in the network were detected as low level tonic signaling. It appears that the mere presence of p53 or CD95 can affect expression levels of miRNAs, although stimulation through CD95 did not have a major effect on the expression of either let-7 or miR-34 (data not shown). Alternatively, up- or downregulation of CD95 may select for cells with a certain miRNA profile. In any case, new methods will have to be developed to monitor and model weak signaling events, which in combination may be critical in determining cell fate.

### The presence of CD95 affects the expression of let-7

**Figure 6:**
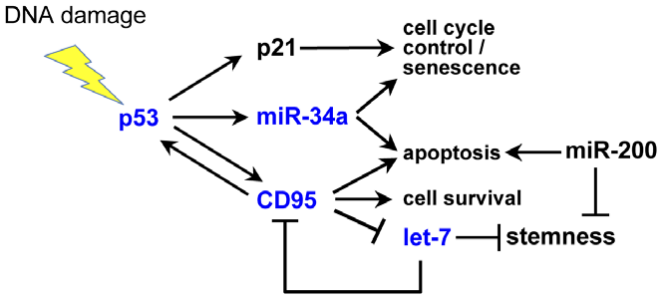
Model of proposed regulatory network. The three signaling branches of p53 leading to opposing outcomes of p53 activation. Experiments in this paper suggest different activation thresholds for p21 and miR-34a upon altering CD95 expression as p21 was equally efficiently induced despite modulation in CD95, whereas miR-34a was responsive to CD95 changes. Nonapoptotic activities of CD95 mentioned in this figure were discussed in recent reviews [77], [78].

That CD95 (and p53) negatively correlates with expression of let-7 can be explained by a model in which both CD95 and p53 are part of a regulatory network. Our data on the connection between p53 and let-7 are consistent with a recent report describing an inhibitory role for p53 in HCT116 cells in which let-7a and let-7b were suppressed upon upregulation of wt p53 induced by γ-irradiation. The suggested mechanism for this inhibition involves direct binding of p53 to an enhancer in the promoter sequence shared by these two let-7 family members [55]. This, however, does not explain the negative correlation between let-7c and both p53 and CD95. Future studies are aimed at determining the mechanism of suppression of let-7 by p53 and CD95. Interestingly, an inverse connection between let-7 and CD95 has been reported. let-7 miRNAs are able to directly target CD95 mRNA causing its degradation and a functional desensitization to CD95-mediated apoptosis [56]. Direct evidence of a negative feedback loop between let-7 and CD95 in multiple cancer cell lines has also been reported [21]. A negative feedback loop was suggested because of a modest reduction in the protein levels of the miRNA processing enzyme Dicer upon stimulation with an anti-CD95 mAb. Based on these data we now formulate a hypothesis that links all of these players, CD95, p53, miR-34a, and let-7, in a regulatory network (**Figure 6**).

### A model for the role of the CD95/let-7/p53/miR-34a regulatory network and its potential relevance in cancer stem cells

We have noticed that each component of this novel network has crucial functions in the generation or maintenance of cancer stem cells (CSCs), which suggests that the network could be involved in the activity of CSCs.

#### Let-7

CSCs have gained much interest as a likely mechanistic explanation for cancer progression, tumor heterogeneity, emergence of aggressiveness, and drug resistance [16], [57], [58]. A number of miRNAs have been shown to participate in the generation and maintenance of CSCs. These include, among others, the miR-17-92 cluster [59]. In contrast, certain miRNAs are not found in CSCs, and their expression is inconsistent with stemness. Not surprisingly, these miRNAs often have tumor suppressive activities. Such miRNAs include the let-7 [20], miR-200 [60], and miR-34 families of miRNAs [61]–[64]. The let-7 miRNAs are possibly the best studied family of stemness regulating miRNAs. Numerous let-7 targets are directly involved in maintenance or induction of stemness. Downregulation of let-7 following chemotherapeutic treatment has been shown to increase stemness and tumorigenicity of breast cancer cells through regulation of multiple targets including the let-7 targets c-Myc, Ras, and HMGA2 [65].

#### p53 and miR-34

In embryonic stem cells, the function of p53 differs from that in normal tissues. ES cells are unable to arrest in G1 upon DNA damage [66], yet in these cells maintenance of genomic integrity is utterly crucial. This is normally achieved by the action of p53 to decrease Nanog and LIF expression, which are required for self-renewal. Taking into account the role of miR-34 as an effector for p53, it is not surprising that miR-34 also participates in protecting both stem cells themselves and the organism from pluripotent cells that have turned rogue. Induction of pluripotency by Oct24, Sox2, Klf4, and c-Myc in mouse embryonic fibroblasts induces all three miR-34 species to cooperatively inhibit reprogramming by repressing Nanog, Sox2, and N-myc [63]. It is likely that the activities of p53 and miR-34 in stem cells also affect CSCs. In fact, transfected pre-miR-34a in prostate cancer cell lines suppressed their stem-like phenotype and decreased tumor load in a xenograft model. Loss of stemness was shown to be due to miR-34a directly targeting and repressing CD44, a widely used marker for stem cells [64]. In addition, pancreatic cancer cells frequently display miR-34 loss due to epigenetic silencing by methylation. Inhibition of methylation and/or histone deacetylase inhibitor in pancreatic cancer stem cells caused marked upregulation of miR-34a and induced both cell cycle arrest and apoptosis [62]. Similar results were obtained when miR-34a was restored by exogenous miRNAs (pre-miR transfection or lentiviral expression) in another pancreatic cancer model resulting in an almost 90% reduction in tumor-initiating CD44+ stem cells [59].

#### CD95

Lately, some interesting data have begun to emerge implicating CD95 in the maintenance and function of stem cells. In 2004 Richards et al. [67] identified CD95 as one of 28 genes upregulated in human ES cells. Among other identified genes were pivotal players required for stemness including Lin28, Oct4, SOX2, and Nanog. Expression of CD95 mRNA was shown to decrease upon differentiation in a similar fashion as the validated let-7 target Lin28. The functional role of CD95 in stem cells has been most clearly elucidated in cells of the central nervous system (CNS). The mRNAs for both receptor and ligand are expressed in neurosphere cultures of mouse embryonal fibroblasts [68]. Neuronal progenitor cells are resistant to CD95L-induced apoptosis, and expression of CD95 in these cells protects them from growth factor removal, a highly apoptotic insult [69]. However the role of CD95 in the CNS is not only to protect stem cells from apoptosis, it directly promotes stem cell maintenance. In the adult brain, neurogenesis occurs only in two regions, the subventricular zone and hippocampus, with the latter being the location of the highest CD95 expression in the central nervous system [12], [70]. Signaling via CD95 in hippocampal neurons promotes both neurite branching and their motility, a form of neurogenesis greatly reduced in neurons from either lpr or gld mice [71]. This function of CD95 in normal healthy cells is dependent on the activation of MEK1/2 and Erk-p35 pathways [12], [72]. Similar to its role in liver regeneration [10], [11], CD95 is required in neuronal regeneration following injury, where it promotes repair and repopulation of damaged areas by stem cells. Most convincingly, CD95 was shown to promote both novel neurogenesis and differentiation of adult neural stem cells via the Src/PI3K/AKT/mTOR pathway [12]. Outside the CNS CD95 has been implicated in the maintenance of stem cell-like human T-cells, which are naïve-like stem cells expressing high levels of CD95 [73]. Taken together, these data point at a physiological role for CD95 in promoting stem cell maintenance and function both in vitro and in vivo. We recently reported that the reduction of CD95 or CD95L expression reduced cell growth of tumor cells and also caused cell cycle arrest in certain cells [10]. This finding is consistent with a proposed activity of CD95 as a regulator of stemness in cancer cells as well. While CD95 may directly affect stemness through regulation of stem cell regulator genes, our data suggest that this could occur through its regulation of let-7, which in turn affects the expression of stem cell genes (**Figure 6**).

In summary, we propose that CD95 is part of a novel regulatory network together with p53 and the miRNAs let-7 and miR-34a. We suggest that CD95 suppresses the stemness-inhibitory let-7 family, thereby predisposing cells to possible adverse outcomes to the loss of this crucial maintainer of cellular differentiation. A component of the model is the negative feedback loop as described by Geng at al. [21] in which let-7 targets and decreases CD95 expression. This might subject cells to the loss of differentiation common during early steps of tumorigenesis [14], [20]. In addition, we also postulate a counteracting pathway in which CD95 maintains p53 expression and, indirectly, the expression of miR-34a, providing a substantial protective axis against the loss of let-7. Further studies will be aimed at identifying the mechanism of CD95 promotion of p53 expression and its mechanism of negatively regulating let-7.

## Materials and Methods

### Cell lines

All cells were maintained in a humidified incubator at 37°C with 5% CO_2_. Colon carcinoma cell lines HCT116 wt and HCT116 p53^−/−^ [74] were from Dr. Bert Vogelstein (Johns Hopkins, MD, USA), and were cultured in McCoy's 5A medium (ATCC #30-2007). The renal carcinoma cell line CAKI-1 and the breast cancer cell line MCF7 were obtained from the National Cancer Institute (as part of the NCI60 cell lines) and grown in RPMI 1640 (Mediatech). All cell media were supplemented with 10% FCS (Sigma Aldrich), 2 mM L-Glutamine, 100 U/ml penicillin (Mediatech). Selection of HCT116 p53^−/−^ cells was performed as detailed in [74] with G418 (Santa Cruz) and Hygromycin B (Sigma Aldrich # H3274).

### CD95 knockdown and overexpression

CD95 knockdown was performed as previously described using the validated CD95 specific shRNA #6 [10]. Cells were maintained under selection with 3 µg/ml puromycin (Sigma Aldrich # P9620). CD95 overexpressing MCF7 cells have been described previously [75].

### Etoposide treatments and western blotting

Cells were treated at a confluency of 50–60% with Etoposide (Sigma Aldrich # E1383) or vehicle control DMSO (Sigma Aldrich) for 12 hours with indicated concentrations. SDS PAGE samples were lysed in a modified RIPA lysis buffer (1% SDS, 1% Triton X-100, 1% deoxycholic acid) and quantified using the DC Protein Assay kit (Bio-Rad). Samples (20 µg protein) were resolved on 12% SDS PAGE gels, transferred to nitrocellulose membrane (Amersham Pharmacia Biotech or Protran, Whatman), blocked with 5% milk-PBST or 2% BSA-PBST, and incubated in primary antibody diluted in blocking solution at 4°C overnight. Antibodies used were: CD95 (C-20, Santa Cruz), p53 (DO-1, Santa Cruz), p21 (DCS60, Cell Signaling Technology), c-Met (clone 25H2, Cell Signaling Technology), and β-actin (AC40, Sigma Aldrich #A3853) along with appropriate HRP-conjugated secondary antibodies (Southern Biotech). Detection was performed using the ECL method (Amersham Pharmacia Biotech) and developed using either X-ray film (Kodak) (**Figures 2A** and **4A**) or chemiluminescence imager G:BOX Chemi XT4 from Synoptics (Frederick MD, USA) (**Figures 2B** and **4B**). Quantifications of band intensities were performed by either using Image-J (version 1.44) or the Genesys v.1.1.2 software (Syngene). In all quantifications relative expression levels normalized to the expression of β-actin in the same lane.

### Cell death assay and surface stainings

Cells were reverse transfected in 6-well plates with 100 nM of either scrambled negative control #2 precursor miRNA or pre-miR-34a oligonucleotides (Ambion) using siPORT NeoFX (Ambion) following the manufacturer's protocol. Three days later cells were split and after attachment over night collected for surface stainings or treated with 1 µg/ml LzCD95L for 18 hrs [22] followed by PI staining. Cells were then transferred to Eppendorf tubes, pelleted and resuspended in 100 µl Nicoletti-buffer (0.1% sodium citrate, 0.1% Triton X-100, 50 µg/ml propidium iodide) and incubated in the dark at 4°C for 4 hours. The percentage of dead cells in triplicate samples as indicated by quantification of cells with sub-G1 DNA content was quantified using a BDLSRFortessa (Becton-Dickinson) and analyzed with FlowJo (Treestar Inc). CD95 surface staining was performed as previously described [10]. To surface stain for CD44 cells were collected into 1 ml of media and stained with either anti-CD44-APC antibody (clone IM7, eBioscience) or isotype control (IgG2b-APC) and analyzed on a BDLSRFortessa and with FlowJo. To inhibit caspase activity cells were pretreated for 1 hour before addition of LzCD95L with 20 µM zVAD-fmk (Enzo LifeSciences).

### Quantitative real-time PCR and miRNA expression data

Total RNA was extracted using the miRNeasy Mini kit (Qiagen) following the manufacturer's protocol. For mRNA expression, 1 µg of RNA was reverse transcribed using random primers included in the High-capacity reverse transcription kit (Applied Biosystems) and cDNAs were quantitated using specific primers from applied Biosystems for GAPDH and p21. For miRNA quantitation 30–60 ng of RNA was reverse transcribed with the High-capacity cDNA reverse transcription kit (Applied Biosystems) and cDNA was quantitated using primers for RNUB6, let-7c, miR-34a, miR-34b, and miR-34c. Results were normalized to GAPDH (mRNA expression) or RNUB6 (miRNA expression).

### miRNA data and COMPARE analyses

The real-time PCR data (ct values) for 208 individual miRNAs were previously described [44], [76], and expression data for each miRNA can be found at http://dtp.nci.nih.gov/mtargets/download.html as WEB_DATA_ISRAEL_MIR.ZIP. To identify miRNAs that correlated with the sensitivity of NCI60 cells to CD95-mediated apoptosis, cell lines were grouped into cells moderately to highly sensitive to CD95-mediated apoptosis (A498, ACHN, BT-549, CAKI-1, CCRF-CEM, EKVX, HCT-116, HCT-15, HOP-92, IGROV1, KM12, LOXIMVI, NCI-H226, NCI-H322M, NCI-H460, SF-295, SR, T-47D, TK-10, UACC-62, UO-31) and completely resistant to CD95-mediated apoptosis (786-0, A549/ATCC, COLO205, DU-145, HCC-2998, HL-60(TB), HOP-62, HS578T, HT29, K-562, M14, MCF7, MDA-MB-231/ATCC, MDA-MB-435, MOLT-4, NCI/ADR-RES, NCI-H23, NCI-H522, OVCAR-3, OVCAR-4, OVCAR-5, OVCAR-8, PC-3, RPMI-8226, SF-268, SF-539, SK-MEL-2, SK-MEL-28, SK-MEL-5, SK-OV-3, SN12C, SNB-19, SNB-75, SW-620, U251, UACC-257) as previously determined [22]. To determine the correlation between miRNA expression and sensitivity to TRAIL induced apoptosis the NCI60 cells were tested for sensitivity to leucine-zipper (Lz) TNF-related apoptosis-inducing ligand (TRAIL). Cells plated in 96 well plates and subjected to 1 µg/ml LzTRAIL. After 18 hours cell death was scored by cell typical morphological changes such as membrane blebbing and/or lifting off the plate. The following cell lines were found to be moderately to highly sensitive to TRAIL induced apoptosis (BT-549, CAKI-1, CCRF-CEM, TK-10, EKVX, DU-145, UACC-257, NCI-H522, OVCAR-4, PC-3, SF-539, HCT-116, HCT-15, HOP-92, KM-12, LOX-IMVI, NCI-H226, NCI-H460, SR, A498, NCI-H322M, SF-295, UACC-62, COLO-205, HOP62, HS578, M14, MDA-MB-231, OVCAR-3, OVCAR-5, RPMI-8226, SW-620, U251) or completely resistant to CD95-mediated apoptosis (IGROV-1, T47D, UO-31, A549, MCF7, MDA-MB-435, MOLT-4, NCI-ADR-RES, NCI-H23, OVCAR-8, SK-MEL-5, SKOV-3, 786-0, HCC-2998, HL-60, HT-29, K562, SF-268, SK-MEL-2, SK-MEL-28, SN-12, SNB-19, SNB-75). For the analysis of let-7d expression shown in **Figure 5A** apoptosis resistant Type I/SC1 (SNB-19, A549, NCI-ADR-RES, OVCAR-3, OVCAR-4, U251, OVCAR-8, HOP-62, SN-12, OVCAR-5, RXF393, MDA-MB-231, HS578, EKVX, DU-145, 786-0, SF-268, SNB-75, SF-539, PC-3) and Type II/SC2 cells (K562, UACC-257, SW-620, COLO-205, SKOV-3, SK-MEL-28, SK-MEL-2, MOLT4, SK-MEL-5, NCI-H23, NCI-H522, MDA-MB-435, HT-29, MCF7, MALME-3M, M14, HL-60) were used as recently described. In a third analysis, miRNAs were identified that best correlated with the ability of NCI60 cells to undergo G1 arrest when γ-irradiated as described [33]. The two groups of cells were cells responsive (A549/ATCC, ACHN, HCT116, IGROV1, LOXIMVI, MALME-3M, MCF7, MOLT-4, NCI-H460, OVCAR-4, SK-MEL-5, SR, UACC-62, UO-31) or unresponsive (786-0, CAKI-1, DU-145, EKVX, HCC-2998, M14, MDA-MB-231/ATCC, MDA-MB-435, MDA-N, NCI/ADR-RES, NCI-H23, NCI-H322M, OVCAR-3, OVCAR-8, PC-3, RXF393, SF-295, SF-539, SK-MEL-28, SN12C, SNB-19, SNB-75, TK-10, U251) when irradiated with 12.6 Gy. Significance of differential expression of miRNAs was determined using unpaired Student's T-test, and miRNAs were ranked according to the p-value. The analyses using gene array data performed in this work covered a total of 130,368 individual gene array probes in the MICROARRAY_ALL target set available at the DTP site (http://dtp.nci.nih.gov/mtweb/search.jsp) distributed over 4 array platforms: STANFORD, GENELOGIC_U95, GENELOGIC_U133, and NOVARTIS representing 18,462 unique genes in the human genome. COMPARE analyses were performed at http://dtp.nci.nih.gov/compare using custom vectors generated using the cell groups listed above. COMPARE analyses were performed using default settings. Gene probes were ranked according to Pearson Correlation Coefficient (PCC).

### Statistical analysis

p-values were calculated using an unpaired Student's t-test.
